# Two-dimensional strain derived parameters provide independent predictors of progression to Chagas cardiomyopathy and mortality in patients with Chagas disease

**DOI:** 10.1016/j.ijcha.2022.100955

**Published:** 2022-01-10

**Authors:** Roberto M. Saraiva, Mauro Felippe F. Mediano, Marcel S.B. Quintana, Gilberto Marcelo Sperandio da Silva, Andréa R. Costa, Andréa S. Sousa, Luiz Henrique C. Sangenis, Fernanda S.N.S. Mendes, Henrique H. Veloso, Sergio S. Xavier, Marcelo T. Holanda, Alejandro Marcel Hasslocher-Moreno

**Affiliations:** Evandro Chagas National Institute of Infectious Diseases, Oswaldo Cruz Foundation, Av. Brasil 4365, Rio de Janeiro, RJ 21040-900, Brazil

**Keywords:** Chagas disease, Mortality, Prognosis, Strain

## Abstract

**Background:**

Patients with chronic Chagas disease (CD) cardiomyopathy have a high mortality. We evaluated if two-dimensional (2D) strain (*ε*) parameters provide independent predictors of progression to CD cardiomyopathy and all-cause mortality.

**Methods:**

A total of 408 patients with chronic CD (58.6% women; 53 ± 11 years; clinical forms: indeterminate 34.1%, cardiac 57.6%, digestive 1.2%, cardiodigestive 7.1%) were consecutively included in this single-center prospective longitudinal study. Echocardiographic evaluation included left atrial and left ventricular (LV) function on *ε* analyses. Primary end-point was a composite of all-cause mortality or heart transplant. Secondary end-point was CD progression defined as the occurrence of changes typical of CD in electrocardiogram, sustained ventricular tachycardia, wall motion abnormalities, or heart failure among patients with the indeterminate form at baseline. Multivariable Cox-proportional-hazards regression analyses were performed to test if 2D *ε* parameters were associated with the studied end-points. *P* values *<* 0.05 were considered significant.

**Results:**

The primary end-point occurred in 91 patients after a follow-up of 6.5 ± 2.7 years. CD progression occurred in 26 out of 144 patients without cardiac form at baseline (2.88 cases/100 patient-years). Peak LV circumferential (HR 1.09, 95% CI 1.01–1.18, *P* = .02) and radial (HR 0.97, 95% CI 0.95–0.99, *P* = .007) *ε*, and LV torsion (HR 0.51, 95% CI 0.35–0.74, *P* = .0004) were independent predictors of the primary end-point. Peak LV radial *ε* (HR 0.96, 95% CI 0.93–0.99, *P* = .03) was an independent predictor of CD progression.

**Conclusions:**

Therefore, 2D *ε* derived parameters can be useful for CD progression and mortality prediction.

## 1 Introduction

Chagas disease (CD) is still a major cause of death in endemic countries and an important cause of morbimortality among immigrant populations in non-endemic countries[1]. Currently, from at least 300,000 to up to one million people living in US are estimated to be infected by *Trypanosoma cruzi*[2]. Chronic CD is classified into indeterminate (no evidence of CD-related disease), cardiac, digestive, and cardiodigestive forms[3]. Most patients present the indeterminate form, but up to 30% present the cardiac form[3]. Patients with cardiac form present a high 10-year mortality rate ranging from 10% among the low-risk group to 84% in the high-risk group[4]. There is also a significant excess mortality rate due to CD against non-CD population in both symptomatic and asymptomatic populations[5]. Progression to the cardiac form occurs at a rate around 2% per year[6]. However, few longitudinal studies identified factors associated with risk for CD progression, such as age, sex, and concomitant cardiac diseases[7], [8]. Echocardiography is a very important tool in CD as identifies cardiomegaly, global and regional wall motion abnormalities, left ventricular (LV) aneurysms, systolic and diastolic dysfunction, and intramural thrombi[9]. Moreover, LV systolic dysfunction is the most consistent independent mortality predictor in CD[10]. Other echocardiographic mortality predictors include right ventricular (RV) dysfunction[11], left atrial (LA) volume[12], LV diastolic function[13], and wall motion score index[14]. Two-dimensional (2D) speckle tracking echocardiographic deformation analysis or strain (ε) may provide a better evaluation of cardiac function and new independent mortality predictors in CD. We described that LV ε decreased progressively since stage B of the cardiac form, while LV torsion decreased progressively in all cardiac form stages[15]. Therefore, the study of the independent prognostic value of these parameters in CD is worthwhile. In fact, in patients with HF due to other etiologies, global LV longitudinal *ε* (LV-GLS) has prognostic value independent from LV ejection fraction (EF)[16], [17]. The fine evaluation of LV contractility may also provide prognostic indexes for CD progression as fibrosis is found in patients with CD indeterminate form[18], [19] and considered a hallmark of CD chronic fibrosing myocarditis[20]. Therefore, we investigated in patients with CD if LA, LV, and RV *ε* derived parameters were independently associated with long-term all-cause mortality. We also evaluated if 2D-Doppler and 2D *ε* echocardiographic indexes were associated with CD progression from the indeterminate to the cardiac form.

## 2 Methods

The data that support the findings of this study are available from the corresponding author upon reasonable request.

This is a single-center prospective longitudinal study. Patients with chronic CD aged between 18 and 80 years were consecutively recruited among those followed at Evandro Chagas National Institute of Infectious Diseases, Oswaldo Cruz Foundation, Rio de Janeiro, Brazil, and referred for echocardiograms between March 2010 and February 2014. CD diagnosis was based on positivity in two different serological tests (enzyme-linked immunosorbent assay and indirect immunofluorescence)[15].

From 557 recruited patients, 2 did not consent and 141 were excluded due to concomitant coronary artery disease (n = 14), valvular heart disease (n = 10), hypertensive heart disease (n = 26), congenital heart defect (n = 2), asthma (n = 10), cancer (n = 15), co-infections (n = 4), kidney transplant (n = 1), kidney failure (n = 9), lupus (n = 1), pregnancy (n = 1), inadequate acoustic window (n = 48), and 6 did not return after the index echocardiogram. The final studied population included 408 patients (58.6% women; 53.1 ± 11.1 years).

This study was approved by the institutional review board under number 0059.0.009.000–09 and conformed to standards applied by the Brazilian National Committee for Research Ethics and Resolution 466/2012 of the National Health Council and to the ethical guidelines of the 1975 Declaration of Helsinki. All subjects gave written informed consent before their participation.

Patients were classified according to the Brazilian CD consensus[3] into: indeterminate, digestive, cardiac (stage A: no HF symptoms with isolated changes in the electrocardiogram [ECG]; stage B: no HF symptoms with segmental or global LV systolic dysfunction; stage C: symptomatic HF; stage D: end-stage HF) or cardiodigestive forms. ECG changes that define the presence of the cardiac form are[21]: complete right bundle-branch block (RBBB), associated or not with left anterior fascicular block (LAHB); frequent polymorphous or repetitive ventricular premature beats (VPBs) > 1 by ECG; nonsustained ventricular tachycardia (VT); second- and third-degree atrioventricular block; sinus bradycardia with heart rate < 40 beats/min; sinus node dysfunction; complete left bundle-branch block (LBBB); atrial fibrillation; electric inactive area (characterized by the presence of pathological Q waves in two contiguous leads in the absence of an intraventricular conduction disturbance); and primary T wave changes. Nonspecific (non-defining CD cardiac form) changes in ECG are sinus bradycardia with heart rate ≥ 40 beats/min, low voltage QRS, nonspecific ST-T changes, first-degree RBBB, LAHB, isolated VPBs, and first-degree atrioventricular block[3], [21].

Primary end-point was a composite of all-cause mortality and heart transplant. Patients were followed as previously described[22] until November 2019. Briefly, patients with the indeterminate form underwent medical visits twice a year and an annual ECG, while patients with the cardiac form underwent medical visits at least four times a year and annual ECG and echocardiogram. Whenever patients with the indeterminate form developed symptoms or changes in the ECG indicative of progression to the cardiac form, they were scheduled to undergo echocardiogram, 24-h Holter monitoring, and a visit to the cardiologist. In case of patients with the cardiac form, additional returns to the medical office and additional ECGs, 24-h Holter monitoring exams, and echocardiograms were determined by clinical status, complications and treatment.

Death was classified as sudden, due to stroke, HF, unrelated to CD, or unknown cause. Death was considered sudden when it occurred within 1 h after of symptoms onset, during sleep, or unwitnessed in a previously stable patient[23]. Death was considered due to HF when it occurred associated to a period of worsening HF clinical status. In case of patients that did not return for medical appointments, mortality data were also retrieved from registries of death certificates available at the department of justice of the Rio de Janeiro state (http://www4.tjrj.jus.br/SEIDEWEB/default.aspx).

Secondary end-point was progression to cardiac form among patients with no evidence of cardiac form at baseline. Progression was defined by the occurrence of new changes in ECG used for diagnosis of CD cardiac form[3], [21], diagnosis of sustained VT, new wall motion abnormalities on echocardiography, or diagnosis of HF.

Echocardiograms were performed using a phased-array ultrasound system (Vivid 7, GE Medical Systems, Milwaukee, WI) equipped with M4S phased-array transducer. Cardiac dimensions and Doppler measurements were obtained as recommended[24], [25]. LV EF and maximum LA volume were determined using modified Simpson’s rule with apical 4- and 2-chamber views images. Echocardiograms were reviewed offline and 2D *ε* analyses were performed with Echopac PC workstation software version 108.1.12 (GE Medical Systems). All 2D clips analyzed were acquired at high frame rates (>60 frames/s).

LA *ε* was determined as previously described[22] using apical 4-and 2-chamber views. The onset of the P-wave was used as the reference point, and peak positive global LA*ε* (LAScd), peak negative global LA*ε* (LASct), and the sum of those previous values (LASr) were obtained.

LV-GLS, LV circumferential (LV-GCS), and radial *ε* (LV-GRS), and LV torsion were calculated as previously described[15]. Peak LV-GCS and LV-GRS were the average of the peak average for LV-GCS and LV-GRS obtained at short-axis views at the basal, mid, and apical levels. Peak LV-GLS was the average of the peak average for LV-GLS obtained at 4-, 2- and 3-chamber views. End-systolic (ES) LV-GLS, LV-GRS, and LV GCS were also obtained similarly. ES timing was defined by the automatic aortic valve closure detection algorithm of the EchoPac software. LV twist was defined as the net-difference of LV rotation between apical and basal short-axis planes and LV torsion as the LV twist divided by the end-diastolic LV longitudinal length. LV length was measured from the middle of a straight line drawn between the two opposite sections of the mitral ring to the most distant point at the LV apex contour in apical views[24].

### 2.1 Statistical analysis

Continuous variables were expressed as mean ± standard deviation and categorical variables as absolute and percentage values. Kolmogorov-Smirnov tests provided support that continuous variables were normal, as *P* values were > 0.10. Data between groups were compared using one-way ANOVA followed by Student-Newman-Keuls post-hoc analysis or contingency tables, as appropriate. Separate multivariable Cox-proportional-hazards regression analyses were performed to test if 2D *ε* parameters were associated with the primary end-point and if any echocardiographic parameter was associated with CD progression. In case of the primary end-point, multivariable analyses were adjusted for parameters reported as independent mortality predictors in CD: age[26], sex[4], low voltage on ECG[4], maximum LA volume[12], LV end-diastolic diameter[27], LV EF[28], ratio of peak early wave diastolic filling velocity to early diastolic mitral annulus velocity (E/E’ ratio)[13], and RV systolic function[11] expressed by peak systolic tricuspid annulus velocity. In case of the secondary end-point, age, sex, and associated cardiac diseases were pointed as associated with CD progression[7], [8]. We also considered that co-morbidities and previous ECG changes might influence CD progression. Therefore, multivariable analysis for secondary end-point prediction were adjusted for age, sex, co-morbidities (hypertension, diabetes mellitus, dyslipidemia, and current smoking), and LAHB. Multicollinearity among the adjustment variables was assessed by variance inflation factor (VIF). Missing data were handled by listwise deletion. Schoenfeld residuals did not reject the proportional assumption of Cox-proportional-hazards regression analysis.

Receiver operating characteristic (ROC) curves were generated to define cut-off values with corresponding sensitivities and specificities for studied end-points prediction. The optimal cutoff obtained for a ROC curve corresponded to the maximum of the Youden index. Areas under the ROC curve (AUC) were compared by pairwise comparison as described by DeLong et al[29]. Cumulative survival curves dichotomized at optimal ROC were constructed using the Kaplan–Meier method and compared using log–rank test.

Calculations were done using MedCalc version 12.5.0.0 (MedCalc Software, Mariakerke, Belgium) and Stata version 13.0 (StataCorp, College Station, TX). The null hypothesis was rejected at *P <* 0.05.

## 3 Results

**Fig. 1:**
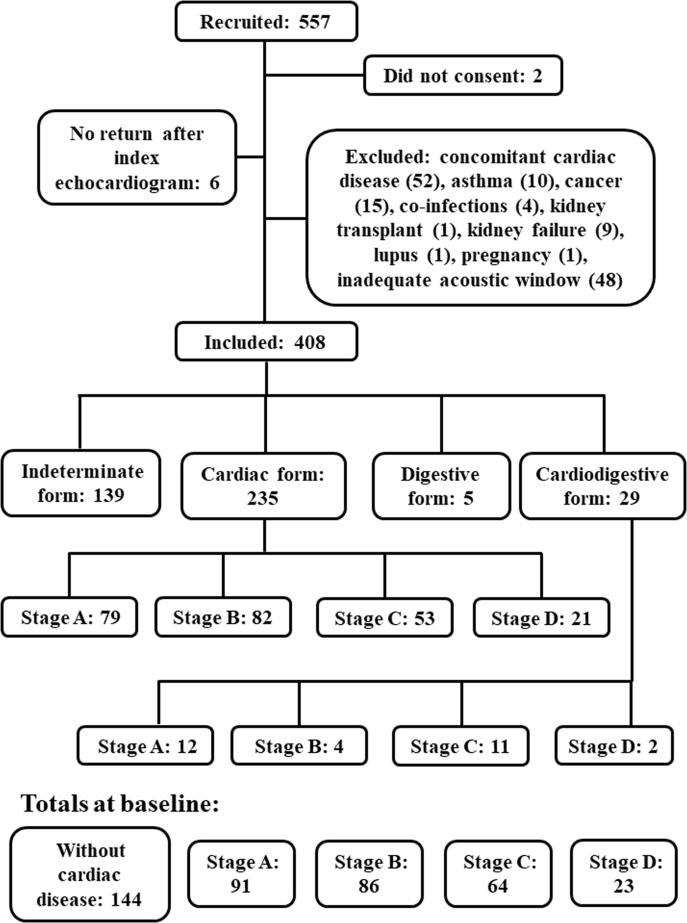
Flow diagram depicting recruitment, exclusion criteria, and studied groups at baseline.

At baseline, 139 patients (34.1%) had the indeterminate form, 235 (57.6%) the cardiac form (stages: A n = 79 [19.4%], B n = 82 [20.1%], C n = 53 [13.0%], D n = 21 [5.1%]), 5 (1.2%) the digestive form, and 29 (7.1%) the cardiodigestive form (stages: A n = 12 [2.9%], B n = 4 [1.0%], C n = 11 [2.7%], D n = 2 [0.5%]). Patients with indeterminate and digestive forms were grouped together in a group named “without evidence of cardiac form” and patients with cardiodigestive form were grouped together with patients with cardiac form according to the stage of the cardiac form (Fig. 1). The clinical, electrocardiographic, and echocardiographic characteristics of these groups are depicted in Table 1. Patients without evidence of cardiac form at baseline were younger than patients with the cardiac form. The body mass index and prevalence of hypertension were lower in patients at the stage D. There was no significant difference in sex distribution and diabetes mellitus prevalence across the groups.

**Table 1:** Clinical and echocardiographic characteristics of studied subjects.

Variable	Without cardiac form n = 144	Stage A n = 91	Stage B n = 86	Stage C n = 64	Stage D n = 23
**Age, years**	49.6 ± 11.5	53.0 ± 10.1*	54.8 ± 9.8*	58.0 ± 9.7*†	56.7 ± 13.0*
**Sex, male**	59 (41.0%)	31 (34.1%)	42 (48.8%)	23 (35.9%)	14 (60.9%)
**Body mass index, g/m^2^**	26.3 ± 4.0	26.2 ± 3.8	25.8 ± 4.1	25.2 ± 4.1	22.1 ± 3.9*†‡§
**Hypertension**	31 (21.5%)	33 (36.3%)*	30 (34.9%)*	15 (23.4%)	2 (8.7%)†‡
**Diabetes mellitus**	6 (4.2%)	4 (4.4%)	3 (3.5%)	6 (9.4%)	3 (13.0%)
**Dyslipidemia**	47 (32.6%)	38 (41.8%)	43 (50%)	21 (32.8%)	7 (30.4%)
**Electrocardiogram**					
RBBB	0	67 (73.6%)*	43 (50%)*†	27 (42.2%)*†	8 (34.8%)*†
LBBB	0	1 (1.1%)	3 (3.5%)	4 (6.2%)	1 (4.3%)
LAHB	9 (6.2%)	45 (49.4%)*	34 (39.5%)*	35 (54.7%)*	10 (43.5%)*
Primary T wave changes	0	26 (28.6%)*	32 (37.2%)*	25 (39.1%)*	8 (34.8%)*
Electric inactive areas	0	1 (1.1%)	2 (2.3%)	5 (7.8%)*	0
Low voltage	5 (3.5%)	3 (3.3%)	7 (8.1%)	5 (7.8%)	3 (13.0%)
Cardiac device	0	10 (11%)*	11 (12.8%)*	12 (18.7%)*	11 (47.8%)*†‡§
Single-chamber pacemaker	0	1 (1.1%)	1 (1.2%)	1 (1.6%)	1 (4.3%)
Dual-chamber pacemaker	0	9 (9.9%)	10 (11.6%)	7 (10.9%)	8 (34.8%)
ICD	0	0	0	3 (4.7%)	2 (8.7%)
CRT	0	0	0	1 (1.6%)	0
**2D echocardiogram**					
LA, cm	3.5 ± 0.4	3.7 ± 0.5*	3.9 ± 0.4*†	4.4 ± 0.6*†‡	4.8 ± 0.6*†‡§
LA volume, ml/m^2^	24.4 ± 6.6	27.2 ± 8.6	30.1 ± 10.5*	45.6 ± 15.1*†‡	62.7 ± 20.4*†‡§
LVd, cm	5.0 ± 0.4	5.1 ± 0.4	5.7 ± 0.7*†	6.6 ± 0.7*†‡	7.3 ± 0.8*†‡§
LVs, cm	3.0 ± 0.4	3.1 ± 0.4	4.1 ± 0.8*†	5.4 ± 0.9*†‡	6.5 ± 0.7*†‡§
LV ejection fraction, %	69.1 ± 6.6	68.0 ± 6.4	54.7 ± 9.1*†	36.7 ± 10.7*†‡	22.7 ± 6.7*†‡§
LV end-diastolic volume, ml/m^2^	51.6 ± 12.9	54.9 ± 10.5	72.8 ± 22.4*†	101.1 ± 30.1*†‡	155.3 ± 45.6*†‡§
LV end-systolic volume, ml/m^2^	16.1 ± 5.5	17.7 ± 5.5	34.2 ± 17.1*†	65.8 ± 27.5*†‡	119.5 ± 33.0*†‡§
LV S’, cm/s	8.7 ± 1.6	8.3 ± 1.8*	6.6 ± 1.3*†	4.8 ± 1.2*†‡	3.5 ± 1.0*†‡§
RV S’, cm/s	13.9 ± 2.3	13.2 ± 2.4*	12.7 ± 2.2*	10.7 ± 2.9*†‡	8.4 ± 2.3*†‡§
TAPSE, mm	24.3 ± 3.7	24.9 ± 4.1	23.3 ± 4.4†	20.6 ± 5.8*†‡	14.1 ± 4.4*†‡§
PASP, mmHg	28.7 ± 4.8	29.9 ± 6.5	31.2 ± 7.9	42.7 ± 12.8*†‡	54.0 ± 15.7*†‡§
E/A ratio	1.3 ± 0.5	1.2 ± 0.5	1.1 ± 0.7*	1.6 ± 0.9*†‡	2.6 ± 1.3*†‡§
E’, cm/s	10.9 ± 3.4	9.5 ± 2.7*	7.2 ± 2.4*†	5.4 ± 1.6*†‡	4.6 ± 1.7*†‡
A’, cm/s	10.1 ± 2.1	10.1 ± 2.3	9.2 ± 2.2*†	6.2 ± 2.4*†‡	3.0 ± 1.2*†‡§
E/E’ ratio	7.3 ± 2.2	8.2 ± 2.6	10.1 ± 3.8*†	17.6 ± 6.9*†‡	23.1 ± 8.8*†‡§
***Strain***					
LASct, %	−13.1 ± 2.8	−12.6 ± 2.9	−12.7 ± 3.5	−7.4 ± 3.1*†‡	−4.2 ± 2.4*†‡§
LAScd, %	15.2 ± 5.0	13.8 ± 4.9*	11.6 ± 5.1*†	8.2 ± 3.8*†‡	3.8 ± 2.1*†‡§
LASr, %	28.3 ± 5.4	26.0 ± 6.3*	24.1 ± 6.0*†	15.1 ± 5.4*†‡	7.8 ± 2.6*†‡§
Peak LV-GLS, %	−19.0 ± 2.4	−18.8 ± 2.4	−14.7 ± 3.1*†	−10.1 ± 3.2*†‡	−5.0 ± 1.7*†‡§
ES LV-GLS, %	−18.1 ± 2.8	−17.7 ± 3.3	−13.5 ± 3.5*†	−9.0 ± 3.3*†‡	−3.6 ± 1.9*†‡§
Peak LV-GCS, %	−19.9 ± 3.4	−19.3 ± 4.1	−14.4 ± 4.5*†	−8.7 ± 3.9*†‡	−5.1 ± 1.9*†‡§
ES LV-GCS, %	−19.1 ± 3.6	−18.2 ± 4.2	−13.6 ± 4.3*†	−8.3 ± 3.8*†‡	−4.8 ± 1.6*†‡§
Peak LV-GRS, %	47.6 ± 12.9	43.5 ± 12.8*	30.9 ± 11.8*†	18.6 ± 12.5*†‡	6.6 ± 5.4*†‡§
ES LV-GRS, %	42.7 ± 12.9	38.5 ± 13.6*	26.0 ± 11.2*†	14.9 ± 11.8*†‡	4.6 ± 4.5*†‡§
Peak Twist, ^0^	12.9 ± 5.7	11.6 ± 5.6	8.7 ± 6.1*†	4.4 ± 4.4*†‡	1.1 ± 3.1*†‡§
Peak Torsion, ^0^/cm	1.6 ± 0.7	1.5 ± 0.7*	1.0 ± 0.7*†	0.5 ± 0.5*†‡	0.1 ± 0.3*†‡§
**Medications**					
Carvedilol	0	0	18 (20.9%)	57 (89.1%)	22 (95.6%)
ACE inhibitor	16 (11.1%)	27 (29.7%)	36 (41.9%)	50 (78.1%)	16 (69.6%)
ARB	1 (0.7%)	2 (2.2%)	6 (7.0%)	10 (15.6%)	6 (26.1%)
Digoxin	0	0	0	23 (35.9%)	11 (47.8%)
Spironolactone	0	0	4 (4.6%)	53 (82.8%)	20 (87%)
Furosemide	0	0	6 (7.0%)	56 (87.5%)	22 (95.6%)
Amiodarone	0	1 (1.1%)	8 (9.3%)	18 (28.1%)	8 (34.8%)

A, peak late wave diastolic filling velocity; A’, peak late diastolic mitral annulus velocity; ACE, angiotensin converting enzyme; ARB, angiotensin receptor blockers; CRT, cardiac resynchronization therapy; E, peak early wave diastolic filling velocity; E’, peak early diastolic mitral annulus velocity; ES, end-systolic; *Ɛ*, strain; GCS, global circumferential *Ɛ*; GLS, global longitudinal *Ɛ*; GRS, global radial *Ɛ*; LA, left atrial; ICD, implantable cardioverter-defibrillator; LAHB, left anterior hemiblock; LASct, peak negative global LA *ε*; LAScd, peak positive global LA *ε*; LASr, total global LA *ε*; LBBB, left bundle branch block; LV, left ventricular; LVd, LV end-diastolic diameter; LVs, LV end-systolic diameter; RBBB, right bundle branch block; RV, right ventricular; PASP, pulmonary artery systolic pressure; S’, peak systolic mitral annulus velocity; TAPSE, tricuspid annular plane excursion.

Values are mean ± SD or n (%).

*p < 0.05 vs. without cardiac form, †p < 0.05 vs. stage A; ‡ p < 0.05 vs. stage B; § p < 0.05 vs. stage C.

There was a gradual increase in chamber size, decrease in LV and RV systolic function, worsening in LV diastolic function across patients with the cardiac form towards the stage D group, who presented severe LV systolic dysfunction (Table 1). LA diameter was larger, and LV and RV peak systolic mitral annulus velocities, and E’ velocity were lower in stage A than patients without evidence of cardiac form.

LA *ε* analyses were feasible in all but three participants. Poor imaging quality was the reason to exclude 18 patients (4.4%) from LV-GLS analysis, 21 patients (5.1%) from LV-GCS and LV-GRS analyses, and 42 patients (10.3%) from LV torsion analysis.

Regarding stain analysis, LA conduit (LAScd) and reservoir (LASr) function were depressed since the initial stages of the cardiac form and further depressed towards the stage D, while the LA contractile function (LASct) was depressed only in those stages with HF (stages C and D). Except for peak LV-GRS, ES LV-GRS, and torsion which were depressed since the stage A of the cardiac form, all other LV strain parameters were depressed since the stage B and further decreased towards stage D group (Table 1).

The intra- and interobserver variabilities for LA and LV *ε* of our group, performed at same equipment and a subgroup of the same population, have already been published[15], [30].

A total of 91 (22.3%) patients presented the primary end-point (89 deaths and 2 heart transplants) during a mean follow-up of 6.5 ± 2.7 years. Patients lost to follow-up (n = 43; 10.5% [without evidence of cardiac form n = 25, stage A n = 9, stage B n = 8, stage C n = 1]) were censored from analysis. The mean LV EF at baseline inside each CD group were similar between patients who were lost or who were not lost to follow-up: without evidence of cardiac form (70 ± 6% vs. 69 ± 7%, *P* = 0.63), stage A of the cardiac form (69 ± 8% vs. 68 ± 6%, *P* = 0.64), and stage B of the cardiac form (53 ± 8% vs. 55 ± 9%, *P* = 0.67).

The cause of death was HF in 44 patients (49.4%), sudden death in 22 patients (24.7%), stroke in 3 patients (3.4%), unrelated to CD in 9 patients (10.1%), and unknown in 11 patients (12.3%). The unrelated to CD deaths were due to: septic shock (n = 3), breast cancer (n = 1), chronic obstructive pulmonary disease (n = 1), electric shock (n = 1), and gastrointestinal complications (n = 3).

Multivariable Cox-proportional-hazards regression analyses models revealed that peak LV-GCS, ES LV-GCS, peak LV-GRS, ES LV-GRS, peak torsion and twist were independent predictors of the primary end-point (Table 2). The full description of these models are depicted in Table S1. The mean and maximum VIF for the variables of adjustment were 2.21 and 3.78, respectively.

**Table 2:** Multivariable models assessing the value of 2D *ε* parameters to predict the primary end-point.

**Variable of Interest**	**HR**	**95% CI**	**P value**^a^	**Model C-index**
LASct, %	1.02	0.94–1.10	0.69	0.89 (0.86–0.92)
LAScd, %	0.98	0.91–1.04	0.46	0.89 (0.86–0.92)
LASr, %	0.97	0.92–1.03	0.32	0.89 (0.86–0.92)
Peak LV-GLS, %	1.04	0.94–1.15	0.40	0.89 (0.86–0.91)
ES LV-GLS, %	1.06	0.97–1.15	0.18	0.88 (0.86–0.91)
**Peak LV-GCS, %**	**1.09**	**1.01**–**1.18**	**0.02**	**0.89 (0.87**–**0.92)**
**ES LV-GCS, %**	**1.09**	**1.01**–**1.18**	**0.02**	**0.89 (0.87**–**0.92)**
**Peak LV-GRS, %**	**0.97**	**0.95**–**0.99**	**0.007**	**0.89 (0.86**–**0.92)**
**ES LV-GRS, %**	**0.97**	**0.95**–**0.99**	**0.01**	**0.89 (0.86**–**0.92)**
**Peak Twist, ^0^**	**0.92**	**0.88**–**0.96**	**0.0004**	**0.89 (0.87**–**0.92)**
**Peak Torsion, ^0^ /cm**	**0.51**	**0.35**–**0.74**	**0.0004**	**0.89 (0.87**–**0.92)**

Abbreviations as in Table 1.

Adjustment variables: age, sex, low voltage, maximum LA volume, LV end-diastolic diameter, LV ejection fraction, E/E’ ratio, and RV S’.

a P value of the variable of interest in the multivariable model

The implantation of cardiac devices may influence the mortality outcome by precluding or postponing cardiovascular death. During this study follow-up, 21 patients received a permanent pacemaker, 21 patients received an implantable cardioverter-defibrillator, and two patients received a cardiac resynchronization therapy device. Among these 44 patients, 20 subsequently presented the primary end-point.

**Fig. 2:**
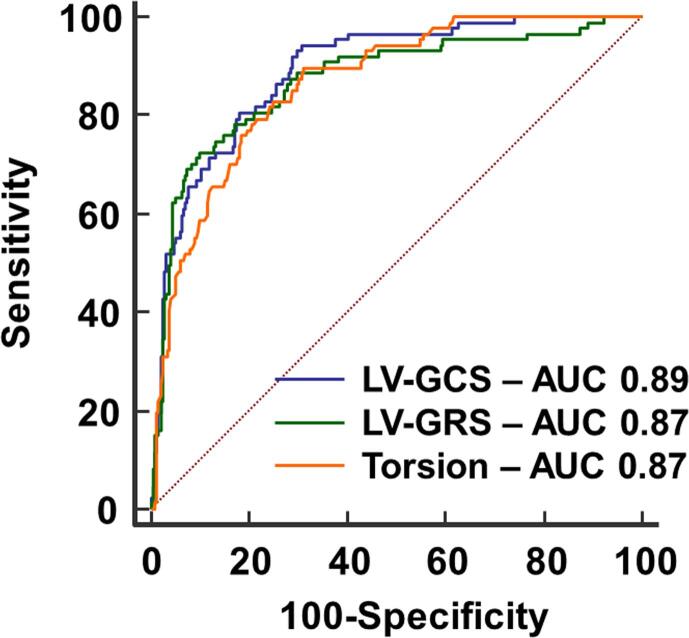
**LV *ε* parameters as predictors of all-cause mortality or heart transplant.** All areas under ROC curves generated for peak LV-GRS, peak LV-GCS, and peak LV torsion were similar. AUC, Area under ROC curve; ε, Strain; LV, Left ventricular; LV-GCS, LV circumferential ε; LV-GRS, LV radial ε; ROC, Receiver operating characteristic.

Optimal cutoff values to predict the primary end-point for peak LV-GRS was 21.9% (AUC 0.87, sensitivity 71.6%, specificity 90.6%, *P* < .0001), for ES LV-GRS was 15.9% (AUC 0.87, sensitivity 69.3%, specificity 92.6%, *P* < .0001), for peak LV-GCS was −15.7% (AUC 0.89, sensitivity 93.2%, specificity 70.9%, *P* < .0001), for ES LV-GCS was −10.3% (AUC 0.89, sensitivity 91.0%, specificity 70.4%, *P* < .0001), for LV twist was 8.1^0^ (AUC 0.86, sensitivity 88.6%, specificity 69.1%, *P* < .0001), and for LV torsion was 1.03^0^/cm (AUC 0.87, sensitivity 89.8%, specificity 69.3%, *P* < .0001). All these variables of interest presented similar AUC (Fig. 2).

**Fig. 3:**
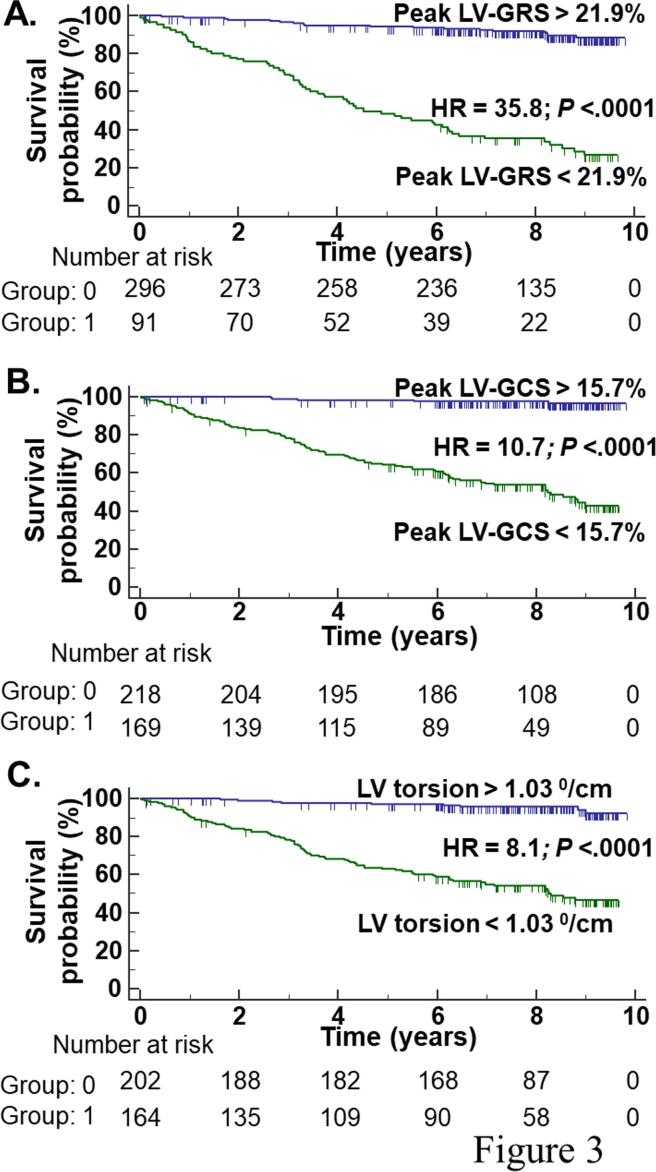
**Survival curves free of all-cause mortality or heart transplant.** Kaplan-Meier curve of the combined end-point free survival according to peak LV-GRS (**A.**), peak LV-GCS (**B.**), and LV torsion (**C.**). LV, Left ventricular; LV-GCS, LV circumferential ε; LV-GRS, LV radial ε.

According to Kaplan-Meier analysis, the primary end-point occurred more frequently in patients with peak LV-GRS ≤ 21.9% (HR = 35.8, 95% CI: 20.8 - 61.6, *P* < .0001; Fig. 3A), peak LV-GCS absolute value ≤ 15.67% (HR = 10.7, 95% CI: 7.0 - 16.5, *P* < .0001; Fig. 3B), and LV torsion ≤ 1.03^0^/cm (HR = 8.1, 95% CI: 5.3 - 12.4, *P* < .0001; Fig. 3C).

The secondary end-point occurred in 26 out of 144 patients without evidence of cardiac form at baseline who progressed to the cardiac form during a mean follow-up of 6.3 ± 2.6 years, which resulted in a 18.05% cumulative progression rate and incidence rate of 2.88 cases/100 patient-years. Time to CD progression was 4.4 ± 2.1 years. Most patients progressed to the stage A (n = 23), while 2 patients progressed to stage B1, and 1 patient to stage C. The criteria for CD progression were new change in ECG (primary T wave changes n = 11, RBBB n = 6, electric inactive areas n = 4, atrial fibrillation n = 1), wall motion changes (n = 2), sustained VT (n = 1), and HF (n = 1). The patient who progressed to stage C also presented primary T wave changes on ECG and severe LV systolic dysfunction on echocardiogram. One patient with wall motion abnormality on echocardiogram also presented 16 months later a RBBB on ECG. One patient whose CD progression criteria was a RBBB, also presented wall motion abnormality 18 months later and finally progressed to HF 2.5 years after RBBB diagnosis.

Multivariable Cox-proportional-hazards regression analyses revealed that peak systolic mitral annulus velocity (LV S’), E’ velocity, and peak LV-GRS were independent predictors of CD progression (Table 3). The mean and maximum VIF for the variables of adjustment were 1.12 and 1.27, respectively.

**Table 3:** Multivariable models assessing the value of echocardiographic parameters to predict CD progression among patients without evidence of cardiac form at baseline.

**Variable of Interest**	**HR**	**95% CI**	**P values**	**Model C-index**
LA, cm	1.36	0.49–3.79	0.56	0.64 (0.53–0.74)
LA volume, ml/m^2^	1.04	0.98–1.10	0.18	0.65 (0.56–0.74)
LVd, cm	1.01	0.36–2.82	0.98	0.64 (0.54–0.74)
LVs, cm	1.87	0.63–5.53	0.26	0.64 (0.53–0.75)
LV ejection fraction, %	0.97	0.92–1.03	0.35	0.65 (0.54–0.75)
LV end-diastolic volume, ml/m^2^	1.02	0.99–1.05	0.25	0.66 (0.57–0.76)
LV end-systolic volume, ml/m^2^	1.07	1.00–1.15	0.06	0.65 (0.55–0.75)
**LV S’, cm/s**	**0.66**	**0.48–0.92**	**0.01**	**0.68 (0.57**–**0.79)**
RV S’, cm/s	0.93	0.77–1.12	0.45	0.66 (0.54–0.78)
TAPSE, mm	0.99	0.88–1.12	0.99	0.64 (0.54–0.74)
E/A ratio	0.53	0.15–1.81	0.53	0.64 (0.53–0.75)
**E’, cm/s**	**0.75**	**0.62–0.91**	**0.004**	**0.69 (0.58**–**0.80)**
A’, cm/s	0.90	0.71–1.13	0.36	0.64 (0.58–0.80)
E/E’ ratio	1.14	0.92–1.41	0.21	0.66 (0.56–0.77)
S/D ratio	0.65	0.18–2.28	0.50	0.64 (0.54–0.75)
Ar, cm/s	1.01	0.96–1.06	0.74	0.65 (0.55–0.75)
***Strain***				
LASct, %	1.08	0.91–1.28	0.35	0.65 (0.53–0.77)
LAScd, %	1.01	0.92–1.12	0.76	0.64 (0.54–0.74)
LASr, %	0.99	0.90–1.08	0.77	0.64 (0.54–0.75)
Peak LV-GLS, %	1.04	0.86–1.26	0.66	0.62 (0.49–0.75)
ES LV-GLS, %	0.98	0.84–1.15	0.84	0.64 (0.54–0.75)
Peak LV-GCS, %	0.90	0.79–1.02	0.09	0.67 (0.57–0.78)
ES LV-GCS, %	0.90	0.80–1.02	0.10	0.67 (0.56–0.77)
**Peak LV-GRS, %**	**0.96**	**0.93–0.99**	**0.03**	**0.70 (0.61**–**0.79)**
ES LV-GRS, %	0.97	0.94–1.00	0.07	0.69 (0.60–0.78)
Peak Twist, ^0^	1.02	0.94–1.11	0.56	0.64 (0.53–0.76)
Peak Torsion, ^0^/cm	1.09	0.59–2.01	0.79	0.65 (0.53–0.76)

Abbreviations as in Table 1.

Adjustment variables: age, sex, hypertension, diabetes mellitus, dyslipidemia, smoking, and LAHB.

Optimal cutoff values to predict the secondary end-point for LV S’ was 8.65 cm/s (AUC 0.64, sensitivity 76.9%, specificity 51.7%, *P* = .03), for E’ velocity was 11.9 cm/s (AUC 0.63, sensitivity 80.7%, specificity 41.5%, *P* = .02), and for peak LV-GRS was 41.78% (AUC 0.66, sensitivity 64%, specificity 75.2%, *P* = .01). All these variables of interest presented similar AUC.

**Fig. 4:**
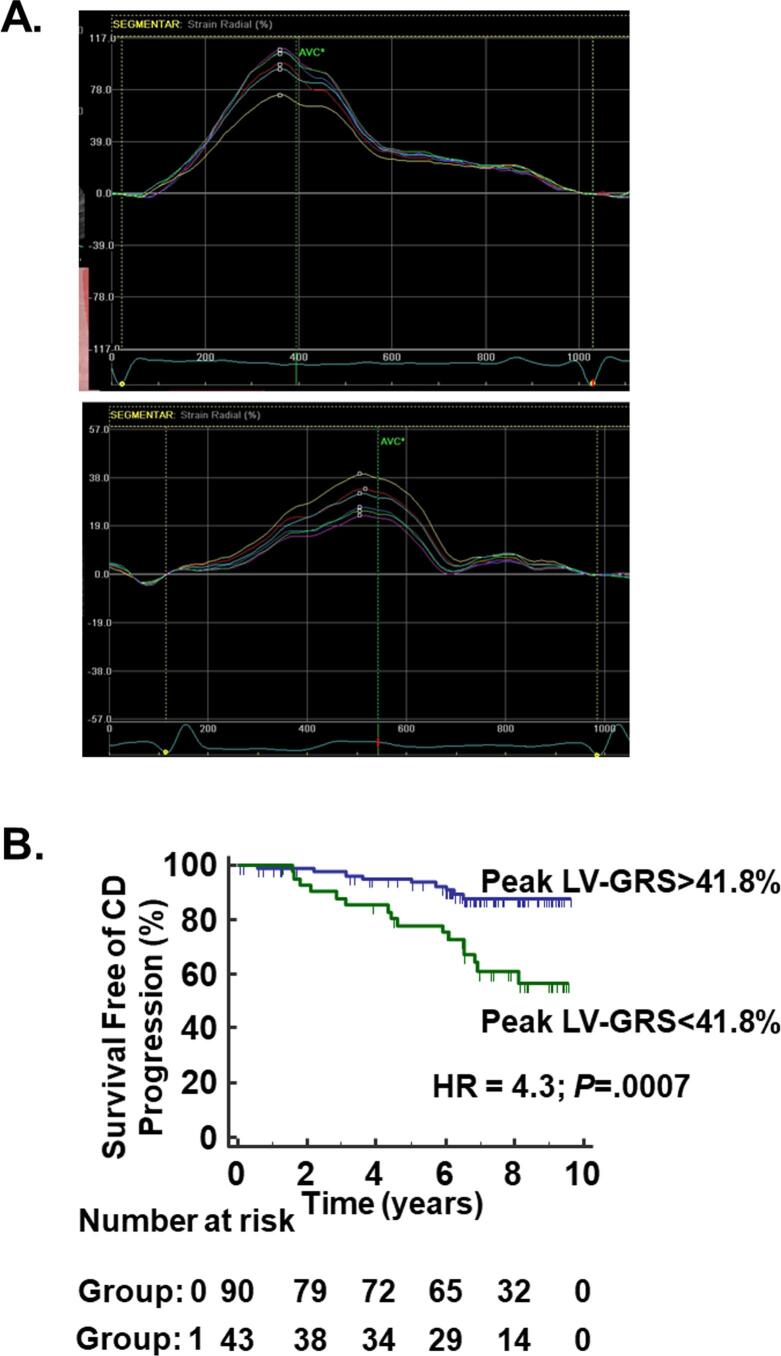
**Chagas disease progression to the cardiac form. A.** Examples of segmental radial strain curves measured by 2D STE from short axis view at the mid level for a patient that did not progress to CD cardiomyopathy after nine years of follow-up **(upper panel)** and a patient that progressed to CD cardiomyopathy after six years of follow-up **(lower panel).** Note the lower radial LV strain of the patient that progressed. **B.** Cumulative survival curve free of Chagas disease progression dichotomized at peak LV-GRS optimal cut-off value (41.8%). CD, Chagas disease; LV-GRS, left ventricular radial ε; STE, speckle tracking echocardiography.

According to the Kaplan-Meier analysis, CD progression was more common in patients with LV S’ ≤8.65 cm/s (HR = 2.6, 95% CI: 1.2–5.6, *P* = .01) and LV-GRS ≤ 41.8% (HR = 4.3, 95% CI: 1.8–9.9, *P* = .0007; Fig. 4).

## 4 Discussion

As far as we know, our paper is the first to evaluate the independent long-term prognostic value of 2D *ε* parameters for mortality prediction in a large sample of patients with chronic CD. Our paper also evaluated if echocardiographic parameters can predict CD progression from the indeterminate to the cardiac form of CD. We found that LV-GCS, LV-GRS, and LV torsion were predictors of all-cause mortality or heart transplant independent from age, sex, low voltage on ECG, and 2D Doppler echocardiographic parameters. We also found that LV S’ and E’ tissue Doppler velocities and peak LV-GRS were predictors of CD progression independent from age, sex, co-morbidities, and LAHB.

There are several different classifications for chronic CD. The II Brazilian Consensus on CD adopted in the present study was designed to classify patients with CD cardiac form. It starts from patients with abnormal ECG (stage A)[3]. On the other hand, the classification of the I Latin American Guidelines[31] and American Heart Association[32] classify patients with the indeterminate form as stage A. Other difference is that patients classified as stages A and B by the II Brazilian Consensus[3], are grouped together as stage B in these two other guidelines[31], [32]. Therefore, it is important to be familiar with the specific classification used by each paper before interpreting their results.

In our study, LV-GLS was not an independent mortality predictor in multivariable analysis that included LV EF among the adjusting variables. LV-GLS evaluates the function of longitudinally orientated myofibers, which are located in the subendocardium[33]. However, in most of the patients at earlier stages of CD cardiac form the pattern of cardiac fibrosis is midwall[30], [34], where circumferentially oriented myofibers predominate[33]. Still, LV-GLS was described as an independent predictor of cardiovascular events in patients with HF due to CD or idiopathic dilated cardiomyopathy[35]. However, this last paper included only patients with HF (stages C and D of CD cardiac form) with a mix of different etiologies.

On the other hand, LV-GCS, LV-GRS, and LV torsion were long-term independent mortality predictors in our study. Accordingly, others described in patients with HF that LV-GCS[16], [17] and LV-GRS[16] were independent predictors of a combined end-point that included mortality. Others also described that LV torsion was an independent predictor of admission due to worsening HF in patients with non-ischemic dilated cardiomyopathy[36]. The fact that basal and apical segments are the walls that most frequently present wall motion changes in CD[3], [31] associated with the fact that the most common cardiac fibrosis pattern found in patients at earlier stages of CD cardiac form is midwall[30], [34], may justify the prognostic value of torsion, LV-GCS and LV-GRS found in our study. In fact, LV torsion, peak LV-GRS, and ES LV-GRS were impaired since the first stage of CD cardiac form in our study, while other LV *ε* parameters were impaired only since the stage B of CD cardiac form. Furthermore, others found a significant difference in ES LV-GRS between patients with the indeterminate form and controls[37]. Therefore, those parameters seem to change earlier during the course of CD cardiac form.

The identification of CD progression predictors is still a challenge. The slow pace of CD progression makes necessary large long-term longitudinal studies, as the present study. The 95% confidence limits of the CD progression rate reported by us is within the reported incidence described by Mota et al (2.57/100 patient-years)[38], but slightly higher than the described by others (1.85/100 patient-years[6] and 1.48/100 patient-years[7]). This may be due to the inclusion of new changes in the echocardiogram as a criterion for progression to CD cardiomyopathy. However, except for one patient, all progressors presented new changes in ECG during the study follow-up and echocardiography was also included in CD progression criteria by others[6]. Importantly, although CD progresses slowly overtime, two patients developed HF during the study follow-up which highlights the importance of following patients with CD indeterminate form and the study of CD progression predictors. Few previous studies pointed a higher risk for CD progression associated with age, sex, and associated cardiac diseases[7], [8]. Our study evaluated the association of a throughout echocardiographic evaluation with CD progression. We found that tissue Doppler parameters and LV-GRS were associated with CD progression. It is possible that these parameters have identified patients with subtle myocarditis and fibrosis. Focal fibrosis is found in up to 20% of patients with CD indeterminate form in cardiac MRI[18], [19], and others found that ES LV-GRS was lower in patients with the indeterminate form than controls[37]. The ongoing active myocarditis would be a necessary mechanism for subsequent CD progression. In fact, recently we showed that cardiac fibrosis mass increases over time in patients with CD[34]. However, it is not entirely clear why LV-GRS and not LV-GLS was associated with subsequent CD progression. We believe that the predominant midwall fibrosis pattern in patients at initial stages of cardiac form[30] may contribute for this difference in CD progression predictive value between LV-GRS and LV-GLS. Moreover, LV S’ and E’ velocities and peak LV-GRS were all lower in the first stage of Chagas cardiomyopathy than in patients without evidence of cardiac form which indicate a possible earlier change in these parameters during progression to CD cardiomyopathy. Although a normal ECG excludes CD progression, the identification of predictors of CD progression is of utmost importance as trypanocide treatment in patients with CD indeterminate form decreases the rate of CD progression[39], [40] and the occurrence of CD related clinical events[39], while trypanocide treatment in patients who already present ECG changes (CD cardiac form) was not associated with changes in clinical prognosis[41].

### 4.1 Limitations

Our study limitations include the use of the same software to measure LV and LA *ε* instead of a dedicated software for LA *ε*, use of the onset of the P wave as the reference frame set to zero LA *ε*, and lack of an external validation. Measurement of LA *ε* has other limitations as outlined elsewhere[42]. Other limitation is that non-sustained VT on 24-hour Holter monitoring was not included among the variables of adjustment in the multivariable model. The inclusion of this variable could have had an impact on the final result of the multivariable model as non-sustained VT is a mortality predictor in CD[4]. Other possible limitation is that sudden death might be also related to coronary artery disease as the studied CD population is aging and present co-morbidities. However, all patients with known coronary artery disease at baseline were excluded from analysis and epicardial coronary arteries are angiographically normal in the majority of patients with CD and atypical angina[32].

Multicollinearity is a potential limitation in multivariable analyses, however the VIF results of the variables used to adjust the multivariable models were under 5, which indicates no evidence of a multicollinearity problem[43].

Another possible limitation in survival studies is the incidence of events that preclude the occurrence of the studied end-point. These are considered competing risks[44]. In our study, 44 participants placed a cardiac device during the study follow-up which could have precluded or postponed a cardiac death. However, 20 out of these 44 patients subsequently died. Besides, we were interested to follow the patient even if such a device was placed on him/her as this is a common event in patients with CD cardiac form.

### 4.2 Conclusions

In conclusion, LV *ε* parameters predicted all-cause mortality or heart transplant in patients with CD independent of clinical and 2D-Doppler echocardiographic indexes. This finding allows the design of validation studies in order to further support the incorporation of such parameters in routine evaluation of patients with CD. Tissue Doppler and LV *ε* parameter predicted CD progression from the indeterminate to the cardiac form. The identification of patients with higher risk of CD progression is very important as those may benefit from treatment with trypanocide drugs.

## 5 Grant support

10.13039/501100004586Fundação de Amparo à Pesquisa do Estado do Rio de Janeiro, Brazil [grant numbers E-26/201.561/2014 and E-26/110.176/2014 to Dr Saraiva], and Conselho Nacional de Desenvolvimento Científico e Tecnológico, Brazil [grant numbers 407655/2012–3, 421843/2017–9, and 305088/2013–0 to Dr Saraiva].

## Declaration of Competing Interest

The authors declare that they have no known competing financial interests or personal relationships that could have appeared to influence the work reported in this paper.
