# Supplementary material for: Two-dimensional strain derived parameters provide independent predictors of progression to Chagas cardiomyopathy and mortality in patients with Chagas disease
Source: Int J Cardiol Heart Vasc. 2022 Jan 10;38:100955. doi: 10.1016/j.ijcha.2022.100955 (PMC8826593; doi:10.1016/j.ijcha.2022.100955)
Supplement: Supplementary data 1 [file mmc1.docx]

**Supplemental Table 1**. Predictors of all-cause mortality or heart transplant including 2D-strain parameters in multivariate analysis models.

|  | **Multivariate Analysis** | | | | | | | | | | | |
| --- | --- | --- | --- | --- | --- | --- | --- | --- | --- | --- | --- | --- |
|  | **Model 0**  **n= 401** | | | **Model A – LASct**  **n=391** | | | **Model B - LAScd**  **n=399** | | | **Model C – LASr**  **n=399** | | |
| **Variable** | **HR** | **95% CI** | **P values** | **HR** | **95% CI** | **P values** | **HR** | **95% CI** | **P values** | **HR** | **95% CI** | **P values** |
| Age, years | **1.03** | **1.00-1.05** | **0.015** | **1.03** | **1.01-1.06** | **0.003** | **1.03** | **1.01-1.05** | **0.01** | **1.03** | **1.00-1.05** | **0.03** |
| Sex, male | 0.71 | 0.44-1.14 | 0.13 | 0.84 | 0.51-1.38 | 0.49 | 0.68 | 0.42-1.10 | 0.12 | 0.67 | 0.42-1.09 | 0.11 |
| Low voltage | 0.91 | 0.40-2.05 | 0.81 | 1.11 | 0.42-2.89 | 0.83 | 0.87 | 0.38-1.97 | 0.74 | 0.82 | 0.36-1.86 | 0.63 |
| LA vol, ml/m^2^ | **1.03** | **1.01-1.04** | **0.0006** | **1.04** | **1.02-1.06** | **<0.0001** | **1.03** | **1.01-1.05** | **0.0002** | **1.03** | **1.01-1.04** | **0.0005** |
| LV ejection fraction, % | **0.95** | **0.92-0.97** | **<0.0001** | **0.96** | **0.93-0.98** | **0.001** | **0.95** | **0.92-0.97** | **0.0001** | **0.95** | **0.93-0.98** | **0.0002** |
| LVd, cm | **1.51** | **1.01-2.25** | **0.04** | 1.37 | 0.90-2.07 | 0.14 | 1.42 | 0.94-2.13 | 0.09 | 1.40 | 0.93-2.11 | 0.10 |
| E/E’ ratio | 0.97 | 0.94-1.01 | 0.18 | 1.00 | 0.96-1.05 | 0.87 | 0.97 | 0.93-1.01 | 0.12 | 0.97 | 0.93-1.01 | 0.10 |
| RV S’, cm/s | 0.99 | 0.91-1.09 | 0.88 | 0.99 | 0.90-1.09 | 0.92 | 1.00 | 0.91-1.09 | 0.94 | 1.01 | 0.92-1.11 | 0.84 |
| **Variable of Interest** | - | - | - | 1.02 | 0.94-1.10 | 0.69 | 0.98 | 0.91-1.04 | 0.46 | 0.97 | 0.92-1.03 | 0.32 |

Abbreviations as in Table 1.

**Supplemental Table 1**. Cont.

|  | **Multivariate Analysis** | | | | | | | | | | | |
| --- | --- | --- | --- | --- | --- | --- | --- | --- | --- | --- | --- | --- |
|  | **Model D – Peak** LV-GLS  **n=385** | | | **Model E – ES** LV-GLS  **n=385** | | | **Model F –** Peak LV-GCS  **n=381** | | | **Model G –** ES LV-GCS  **n=381** | | |
| **Variable** | **HR** | **95% CI** | **P values** | **HR** | **95% CI** | **P values** | **HR** | **95% CI** | **P values** | **HR** | **95% CI** | **P values** |
| Age, years | **1.02** | **1.00-1.05** | **0.02** | **1.02** | **1.00-1.04** | **0.02** | **1.03** | **1.01-1.05** | **0.01** | **1.03** | **1.01-1.05** | **0.01** |
| Sex, male | 0.68 | 0.42-1.11 | 0.13 | 0.68 | 0.41-1.10 | 0.12 | 0.67 | 0.41-1.09 | 0.11 | 0.67 | 0.41-1.10 | 0.12 |
| Low voltage | 0.85 | 0.38-1.93 | 0.70 | 0.83 | 0.37-1.88 | 0.66 | 0.84 | 0.37-1.92 | 0.68 | 0.86 | 0.38-1.96 | 0.72 |
| LA vol, ml/m^2^ | **1.03** | **1.01-1.04** | **0.0004** | **1.03** | **1.01-1.04** | **0.0003** | **1.02** | **1.01-1.04** | **0.002** | **1.02** | **1.01-1.04** | **0.002** |
| LV ejection fraction, % | **0.95** | **0.92-0.99** | **0.005** | **0.96** | **0.93-0.99** | **0.005** | **0.97** | **0.94-0.99** | **0.03** | **0.97** | **0.94-0.99** | **0.02** |
| LVd, cm | 1.46 | 0.97-2.20 | 0.07 | 1.50 | 0.99-2.25 | 0.05 | 1.34 | 0.88-2.04 | 0.18 | 1.36 | 0.89-2.06 | 0.15 |
| E/E’ ratio | 0.97 | 0.93-1.01 | 0.12 | 0.97 | 0.93-1.00 | 0.08 | 0.98 | 0.94-1.02 | 0.36 | 0.98 | 0.94-1.02 | 0.37 |
| RV S’, cm/s | 1.00 | 0.92-1.10 | 0.91 | 1.01 | 0.92-1.10 | 0.87 | 1.00 | 0.92-1.10 | 0.94 | 1.00 | 0.91-1.10 | 0.97 |
| **Variable of Interest** | 1.04 | 0.94-1.15 | 0.40 | 1.06 | 0.97-1.15 | 0.18 | **1.09** | **1.01-1.18** | **0.02** | **1.09** | **1.01-1.18** | **0.02** |

Abbreviations as in Table 1.

**Supplemental Table 1**. Cont.

|  | **Multivariable Analysis** | | | | | | | | | | | |  |
| --- | --- | --- | --- | --- | --- | --- | --- | --- | --- | --- | --- | --- | --- |
|  | **Model H –** Peak LV-GRS  **n=381** | | | **Model I –** ES LV-GRS  **n=381** | | | **Model J –** Twist  **n=360** | | | **Model K –** Torsion  **n=360** | | | |
| **Variable** | **HR** | **95% CI** | **P values** | **HR** | **95% CI** | **P values** | **HR** | **95% CI** | **P values** | **HR** | **95% CI** | **P values** | |
| Age, years | **1.03** | **1.01-1.05** | **0.01** | **1.03** | **1.00-1.05** | **0.02** | **1.02** | **1.00-1.05** | **0.03** | **1.02** | **1.00-1.05** | **0.02** | |
| Sex, male | 0.74 | 0.46-1.21 | 0.23 | 0.77 | 0.47-1.25 | 0.29 | 0.80 | 0.49-1.29 | 0.36 | 0.79 | 0.49-1.28 | 0.34 | |
| Low voltage | 0.85 | 0.37-1.93 | 0.70 | 0.89 | 0.39-2.01 | 0.77 | 1.02 | 0.45-2.30 | 0.97 | 1.00 | 0.44-2.27 | 0.99 | |
| LA vol, ml/m^2^ | **1.02** | **1.01-1.04** | **0.004** | **1.02** | **1.01-1.04** | **0.003** | **1.02** | **1.01-1.04** | **0.008** | **1.02** | **1.01-1.04** | **0.006** | |
| LV ejection fraction, % | **0.96** | **0.94-0.99** | **0.008** | **0.96** | **0.94-0.99** | **0.002** | **0.96** | **0.93-0.98** | **0.001** | **0.96** | **0.94-0.98** | **0.001** | |
| LVd, cm | 1.38 | 0.92-2.01 | 0.12 | 1.39 | 0.92-2.10 | 0.11 | 1.43 | 0.96-2.14 | 0.08 | 1.43 | 0.96-2.14 | 0.08 | |
| E/E’ ratio | 0.98 | 0.94-1.02 | 0.31 | 0.98 | 0.94-1.02 | 0.29 | 0.98 | 0.94-1.02 | 0.35 | 0.98 | 0.94-1.02 | 0.33 | |
| RV S’, cm/s | 0.99 | 0.90-1.08 | 0.78 | 0.99 | 0.91-1.08 | 0.80 | 0.98 | 0.90-1.08 | 0.75 | 0.98 | 0.90-1.07 | 0.68 | |
| **Variable of Interest** | **0.97** | **0.95-0.99** | **0.007** | **0.97** | **0.95-0.99** | **0.01** | **0.92** | **0.88-0.96** | **0.0004** | **0.51** | **0.35-0.74** | **0.0004** | |

Abbreviations as in Table 1.
